# Differential Sensitivity of Src-Family Kinases to Activation by SH3 Domain Displacement

**DOI:** 10.1371/journal.pone.0105629

**Published:** 2014-08-21

**Authors:** Jamie A. Moroco, Jodi K. Craigo, Roxana E. Iacob, Thomas E. Wales, John R. Engen, Thomas E. Smithgall

**Affiliations:** 1 Department of Microbiology and Molecular Genetics, University of Pittsburgh School of Medicine, Pittsburgh, Pennsylvania, United States of America; 2 Center for Vaccine Research, University of Pittsburgh School of Medicine, Pittsburgh, Pennsylvania, United States of America; 3 Department of Chemistry and Chemical Biology, Northeastern University, Boston, Massachusetts, United States of America; Universität Erlangen-Nürnberg, Germany

## Abstract

Src-family kinases (SFKs) are non-receptor protein-tyrosine kinases involved in a variety of signaling pathways in virtually every cell type. The SFKs share a common negative regulatory mechanism that involves intramolecular interactions of the SH3 domain with the PPII helix formed by the SH2-kinase linker as well as the SH2 domain with a conserved phosphotyrosine residue in the C-terminal tail. Growing evidence suggests that individual SFKs may exhibit distinct activation mechanisms dictated by the relative strengths of these intramolecular interactions. To elucidate the role of the SH3:linker interaction in the regulation of individual SFKs, we used a synthetic SH3 domain-binding peptide (VSL12) to probe the sensitivity of downregulated c-Src, Hck, Lyn and Fyn to SH3-based activation in a kinetic kinase assay. All four SFKs responded to VSL12 binding with enhanced kinase activity, demonstrating a conserved role for SH3:linker interaction in the control of catalytic function. However, the sensitivity and extent of SH3-based activation varied over a wide range. In addition, autophosphorylation of the activation loops of c-Src and Hck did not override regulatory control by SH3:linker displacement, demonstrating that these modes of activation are independent. Our results show that despite the similarity of their downregulated conformations, individual Src-family members show diverse responses to activation by domain displacement which may reflect their adaptation to specific signaling environments in vivo.

## Introduction

The Src family of non-receptor protein-tyrosine kinases is the largest tyrosine kinase family in the human kinome, with multiple members expressed in virtually every cell type. Three Src-family kinases (SFKs), c-Src, Fyn, and c-Yes, are found in most cell types while the remaining members have more restricted expression patterns, primarily in hematopoietic cell lineages [1]. SFKs regulate multiple cellular processes, including proliferation, survival, differentiation, adhesion and migration [2]. Strict control of SFK activity is essential to normal cellular and tissue homeostasis, while deregulation of SFK activity contributes to malignant transformation. Increased c-Src expression and activity are both observed in many forms of cancer and contribute to tumor cell proliferation, migration, and invasiveness [3].

All SFKs share a high degree of amino acid sequence homology and identical domain organization. The N-terminus of each family member encodes a signal sequence for myristoylation, a lipid modification critical to membrane localization. The myristoylation site is followed by a relatively short unique domain, which is the only non-homologous region across the kinase family. Most SFKs are also palmitoylated within this region, which contributes to membrane targeting [4]. Following the unique region is an SH3 domain, which binds proline-rich sequences that adopt a polyproline type-II (PPII) helical structure, an SH2 domain, which binds phosphotyrosine-containing sequences, an SH2-kinase linker, a bilobed kinase domain, and a C-terminal negative regulatory tail. All SFKs contain two conserved tyrosine phosphorylation sites important for regulation of kinase activity, which serve opposing roles [5]. Phosphorylation of Tyr416 (all residue numbering as per the structure of c-Src; PDB code 2SRC) in the activation loop stabilizes the active conformation of the kinase domain. Conversely, phosphorylation of Tyr527 in the C-terminal tail is required for downregulation of kinase activity. This site is phosphorylated by the separate regulatory kinases Csk and Chk [6].

**Figure 1:**
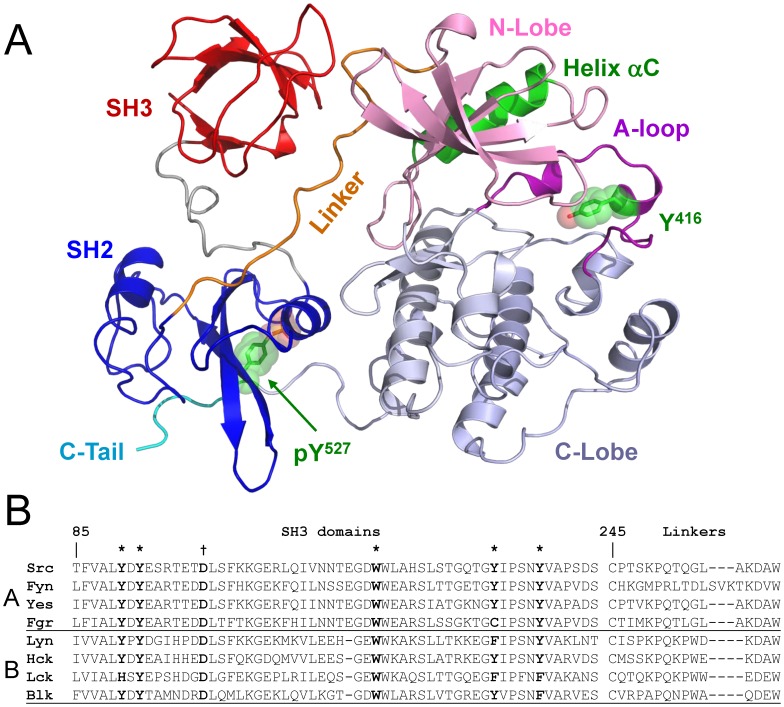
Structural features of Src-family kinases. A) Crystal structure of inactive c-Src (PDB: 2SRC) [8] showing the intramolecular interactions necessary for downregulation of kinase activity. Shown are the SH3 domain (red), 3–2 connector (gray), SH2 domain (blue), SH2-kinase linker (orange), kinase domain (N-lobe, pink; C-lobe, light blue), and the C-terminal pTyr tail (cyan, pTyr527 side chain shown in green). The N-lobe αC-helix is shown in green. The SH3 domain interacts with the PPII helix formed by the linker, while the SH2 domain interacts with the pTyr tail. In the inactive state, the activation loop (purple) adopts a partially helical conformation and the autophosphorylation site (Tyr416) points inward towards the catalytic cleft. B) Sequence alignment of Src-family kinase SH3 domains and SH2-kinase linkers. The Src family can be divided into two subfamilies based on sequence homology as shown (A and B subgroups). Key hydrophobic residues that contribute to the binding surface are highlighted in bold and marked with an asterisk (their positions in the structure of the Src SH3 domain are modeled in Figure 2B). The conserved aspartate residues (Asp99 in c-Src) that contribute to VSL12 peptide binding are also bolded and marked with a †. SFK linker sequences are more diverse and display suboptimal residues at key positions that face the SH3 domain in the inactive state. The positions of linker residues that contact the SH3 domain in the inactive structure of c-Src are modeled in Figure 2B.

Biochemical and structural studies have provided detailed insight regarding the mechanisms that keep SFKs in the inactive state. Both c-Src and the hematopoietic family member Hck have been crystallized in their inactive conformations [7]–[10], revealing two intramolecular interactions essential for downregulation of kinase activity (Figure 1). One interaction involves pTyr527 in the C-terminal tail and the SH2 domain, while the other includes the SH3 domain and the PPII helix formed by the SH2-kinase linker. When both interactions are present, the SH3 and SH2 domains pack against the back of the kinase domain to stabilize the inactive conformation. In the inactive state, helix αC in the N-lobe is rotated away from the active site, preventing a conserved glutamate residue (Glu310 in c-Src) from forming a salt bridge with Lys295, a conserved interaction critical to the activity of c-Src and virtually all other protein kinases [8], [10]. As a consequence, the activation loop adopts a partially helical structure with the autophosphorylation site (Tyr416) pointed inward.

Sequence homology and shared domain organization suggest that the structural basis of kinase downregulation is conserved across all Src-family members. However, distinct mechanisms of kinase activation have been reported for individual SFKs, including displacement of the SH3 domain from the SH2-kinase linker [11], displacement of the C-terminal tail from the SH2 domain [12], displacement of both of these intramolecular interactions [13], and *trans*-phosphorylation of the activation loop tyrosine by another kinase [5]. Further evidence suggests that these activation mechanisms may be unique to individual family members. For example, Hck is activated by the HIV-1 virulence factor Nef, which binds to the Hck SH3 domain. SH3 domain engagement by Nef displaces the Hck linker, leading to kinase activation without displacement of the tail from the SH2 domain [14], [15]. In contrast, both the SH3:linker and SH2:tail interactions are disrupted when c-Src is activated by focal adhesion kinase (FAK), which contains tandem docking sites for the c-Src SH3 and SH2 domains [13].

Other evidence suggests that individual SFKs serve unique and non-redundant roles within the same cell type, despite their close structural homology. Recent work in murine embryonic stem (ES) cells provides a dramatic example of this phenomenon. Although c-Src and its closest phylogenetic relative, c-Yes, are co-expressed in this cell type, c-Src activity drives ES cell differentiation while c-Yes suppresses differentiation and drives self-renewal [16]–[18]. Along similar lines, RNAi-mediated knock-down of c-Yes expression in colon cancer cell lines increased apoptosis and reduced cell migration and tumor growth, while c-Src knock-down yielded a much less dramatic effect on the same phenotypes, suggestive of a dominant role for c-Yes vs. c-Src in colon cancer [19]. These differences in individual functions raise fundamental questions about what mechanisms selectively regulate specific family members, especially in cellular contexts where multiple SFKs are co-expressed.

Biological evidence for diversity in SFK signaling prompted us to investigate whether individual family members are controlled by regulatory domain displacement and autophosphorylation in an equivalent manner. For this study, we focused on four Src family members, c-Src, Lyn, Fyn and Hck, because they are broadly representative of the overall family from a phylogenetic point of view. Using a synthetic SH3-binding peptide, we demonstrate that all four SFKs are susceptible to activation by SH3 domain displacement, although the sensitivity to activation by this mechanism varied widely. In addition, we observed a major difference in sensitivity to activation of c-Src vs. Hck by SH3 domain displacement following autophosphorylation of the activation loop. Overall, these observations suggest that the mode of activation (activation loop phosphorylation vs. regulatory domain displacement) may reflect the evolution of individual SFKs to accommodate specific signaling environments.

## Results

### Structural basis of high affinity VSL12 peptide binding to SFK SH3 domains

To test c-Src, Fyn, Hck, and Lyn for their susceptibility to activation by SH3 domain displacement, we first identified a peptide ligand with similar affinity for each of their SH3 domains. VSL12, a 12-mer peptide with the sequence VSLARRPLPPLP, was originally discovered in a phage-display screen for sequences that bind to the c-Src SH3 domain in the low micromolar range [20]. Subsequent structural studies showed that VSL12 binds to the Src SH3 domain through a polyproline type II helix found in the C-terminal end of the peptide, with the adjacent arginine making contact with an aspartate in the RT-loop of the c-Src SH3 domain [21]. These observations suggest that VSL12 may bind SFK SH3 domains with similar potency, thereby representing a useful ligand to probe the role of the SH3 domain in kinase regulation across the Src family.

**Figure 2:**
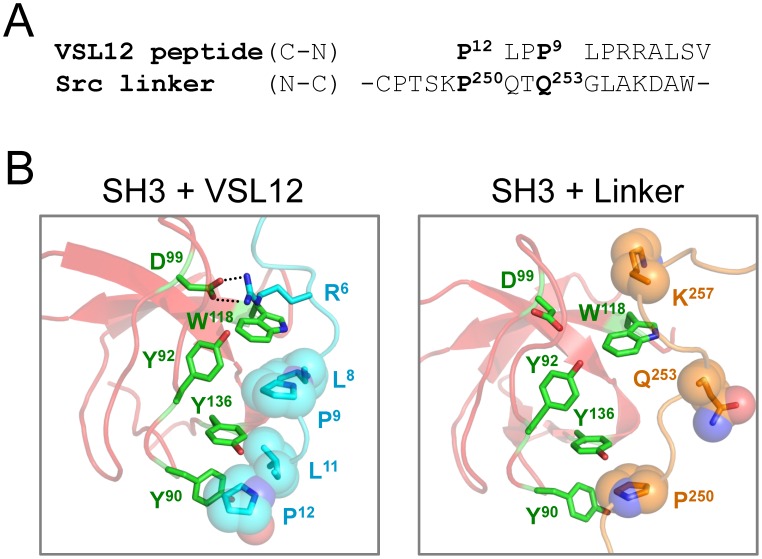
Interaction of the VSL12 peptide with the SH3 domain of c-Src. A) Comparison of the sequence of the SH3-binding peptide VSL12 (*top*) with that of the c-Src SH2-kinase linker (*bottom*). Note that the VSL12 sequence is presented in the C- to N-terminal orientation relative to the linker. B) Comparison of c-Src SH3 domain interaction with the VSL12 peptide and the SH3-kinase linker. The NMR solution structure of the Src SH3 domain (red) with the VSL12 peptide (cyan) is modeled on the left (PDB: 1QWF) [21]. The side chains of the SH3 domain residues that interact with VSL12 are shown in green (tyrosines 90, 92, 136 and Trp118), and interacting VSL12 side chains are shown in cyan (Pro12, Leu11, Pro9, and Leu8). The ionic contact between VSL12 Arg6 and SH3 Asp99 is also shown. The analogous interaction of the Src SH3 domain with the SH2-kinase linker from the inactive structure of c-Src is shown on the right (PDB: 2SRC) [8]. Linker residue Pro250 contributes to SH3 interaction in the P_0_ position of the linker PPII helix, while Gln253 occupies the P_+3_ position and is rotated away from the SH3 surface. The position of Lys257 is also shown; it does not contact Asp99 in this structure.

The amino acid sequence of VSL12 is aligned with the SH2-kinase linker of c-Src in Figure 2A, and structural models of the c-Src SH3 domain bound to VSL12 vs. its own SH2-kinase linker are presented in Figure 2B. Comparison of these structures reveals that VSL12 forms a much more extensive interface with the SH3 domain compared to the natural linker. Proline residues at VSL12 positions 12 and 9, together with adjacent leucine residues (Leu11 and Leu8), occupy grooves formed by conserved SH3 domain residues Tyr90, Tyr92, Trp118, and Tyr136 (see Figure 1B for SH3 domain alignment). In addition, VSL12 has an Arg residue in position 6, which forms a salt bridge with Asp99 in the Src SH3 domain; aspartate is found at this position in all SFK SH3 domains as well (Figure 1B). By comparison, the c-Src SH2-kinase linker forms fewer contacts with the SH3 domain in the context of the overall downregulated structure of c-Src. While the linker does form a PPII helix, only one proline is present in the sequence (Pro250) and packs between SH3 Tyr90 and Tyr 136 in a similar position as Pro12 in the SH3:VSL12 peptide structure (Figure 2B). However, Gln253 occupies the position normally occupied by proline in high-affinity SH3 ligands (Pro9 in VSL12). The long polar side chain of Gln253 is too bulky to fit in the pocket formed by SH3 Tyr92, Trp118, and Tyr136, and points away from the SH3 domain. While a basic residue (Lys257) is present near the C-terminal end of the linker SH3-binding sequence, it does not form a salt bridge with the conserved aspartate (Asp99) in the SH3 domain. These observations suggest that the linker sequence represents a suboptimal SH3 ligand, and that VSL12 may be able to compete with the linker for SH3 binding.

### The VSL12 peptide binds SFK SH3 domains with similar affinity and competes with the linker for near-full-length kinase binding

**Figure 3:**
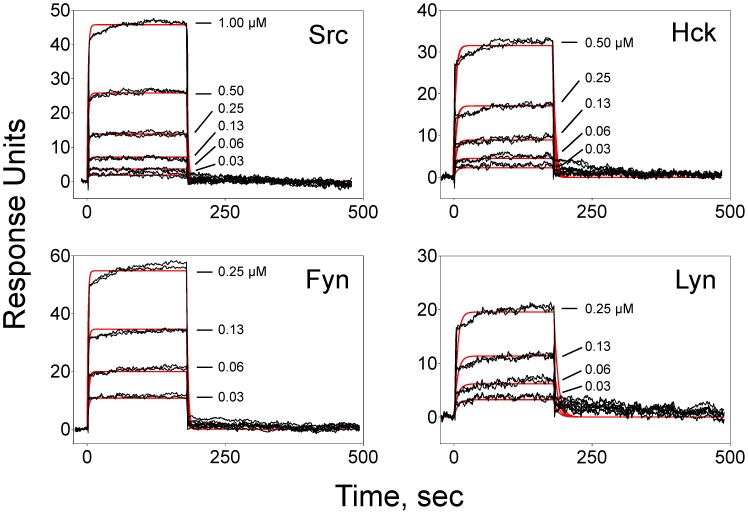
Surface plasmon resonance (SPR) analysis of SH3 interactions with the VSL12 peptide. SPR was used to evaluate VSL12 peptide binding kinetics and affinity for the isolated SH3 domains of c-Src, Hck, Fyn and Lyn as indicated. The biotinylated peptide was immobilized to 80 Response Units (RU) on the surface of a streptavidin (SA) biosensor chip. The recombinant purified Src SH3 domain proteins were flowed past the peptide over the concentration ranges shown. Association was measured for 180 s, followed by a 300 s dissociation phase. Each panel shows a representative sensorgram, with the double-referenced binding data (black traces) fit to a 1∶1 Langmuir binding model (red trace). Kinetic constants derived from this experiment are presented in Table 1.

We first measured the affinity of the isolated SFK SH3 domains for the VSL12 peptide using surface plasmon resonance (SPR). For these experiments, VSL12 was immobilized on a biosensor chip and the recombinant SH3 domains were flowed past the peptide as described under Materials and Methods. Surface densities were kept low to ensure that binding conformed to a 1∶1 Langmuir interaction. As shown in Figure 3, all four SH3 domains bound to VSL12 in a concentration-dependent manner with rapid on-rates, and showed complete dissociation from the peptide following washout. Table 1 summarizes the resulting association (k_a_) and dissociation (k_d_) rate constants as well as the equilibrium dissociation constants derived from them (K_D_). All four SH3 domains bound to VSL12 with low micromolar affinities, consistent with previously published values for the c-Src and Hck SH3 domains [20], [22]. As negative controls, we also performed SPR experiments with the recombinant SH2 domains of all four kinases as well as the kinase domains of c-Src and Hck. As expected, no binding was detected, demonstrating that the VSL12 peptide interacts exclusively with the SH3 domain of each kinase (Table 1).

**Table 1:** Binding Affinities of SFK SH3 Domains for the VSL12 Peptide as Measured by SPR.

Protein	k_a_ (M^−1^s^−1^±SE)	k_d_ (s^−1^±SE)	Kinetic K_D_ (M)	Kinetic χ^2^	Steady-State K_D_ (M)	Steady State χ^2^
Src SH3^a^	1.21×10^5^±0.03×10^5^	4.14×10^−1^±0.08 ×10^−1^	3.42×10^−6^	0.570	4.00×10^−6^	0.260
Fyn SH3^b^	1.06×10^6^±0.03×10^6^	3.72×10^−1^±0.10×10^−1^	3.51×10^−7^	1.890	3.70×10^−7^	1.070
Hck SH3^c^	7.88×10^4^±0.36×10^4^	2.17×10^−1^±0.04×10^−1^	2.76×10^−6^	1.360	2.99×10^−6^	0.390
Lyn SH3^b^	2.12×10^5^±0.10×10^5^	1.37×10^−1^±0.04×10^−1^	6.47×10^−7^	1.850	5.62×10^−7^	0.225
SH2^d^	ND	ND	-	-	-	-
Kinase^d^	ND	ND	-	-	-	-

Analyses were performed with biotinylated VSL12 peptide bound to a streptavidin biosensor chip as described under Materials and Methods. Each protein was flowed past the chip surface over the concentration ranges indicated in the footnotes. Duplicate runs were performed for each concentration. A control cycle of buffer only was subtracted from all concentrations of reference-subtracted curves. To calculate the kinetic K_D_, interaction data were curve-fit using a 1∶1 Langmuir model, with binding constants and chi-squared values calculated using the BIAevaluation software. To calculate the steady state K_D_, the analyte response at equilibrium was plotted against the analyte concentration, and resulting curves were fit with the steady state model in the BIAevaluation software.

a 31.25, 62.5, 125, 250, 500, 1000 nM.

b 31.25, 62.5, 125, 250 nM.

c 31.25, 62.5, 125, 250, 500 nM.

d SH2 domains tested: Src, Hck, Fyn and Lyn; kinase domains tested, Src and Hck. ND, binding not detected with 1 µM protein input.

### Characterization of recombinant SFK-YEEI protein kinase activity in vitro

Characterization of the sensitivity of individual SFKs to SH3-based activation required expression and purification of each Src-family member in the downregulated conformation. Therefore, we used purified recombinant SFK proteins in which the natural C-terminal tails were replaced with the sequence pTyr-Glu-Glu-Ile-Pro (referred to hereafter as SFK-YEEI proteins). This tail modification creates an optimal SH2-binding sequence and has been used in previous structural and enzymatic studies to ensure downregulation of kinase activity through enhanced binding of the tail to the SH2 domain in the absence of Csk co-expression [7], [23]. In addition, the increased affinity of the tyrosine-phosphorylated YEEI tail for the SH2 domain ensured that kinase activation observed in response to VSL12 binding can be attributed to the displacement of the SH3 domain rather than complete disruption of the regulatory apparatus.

**Figure 4:**
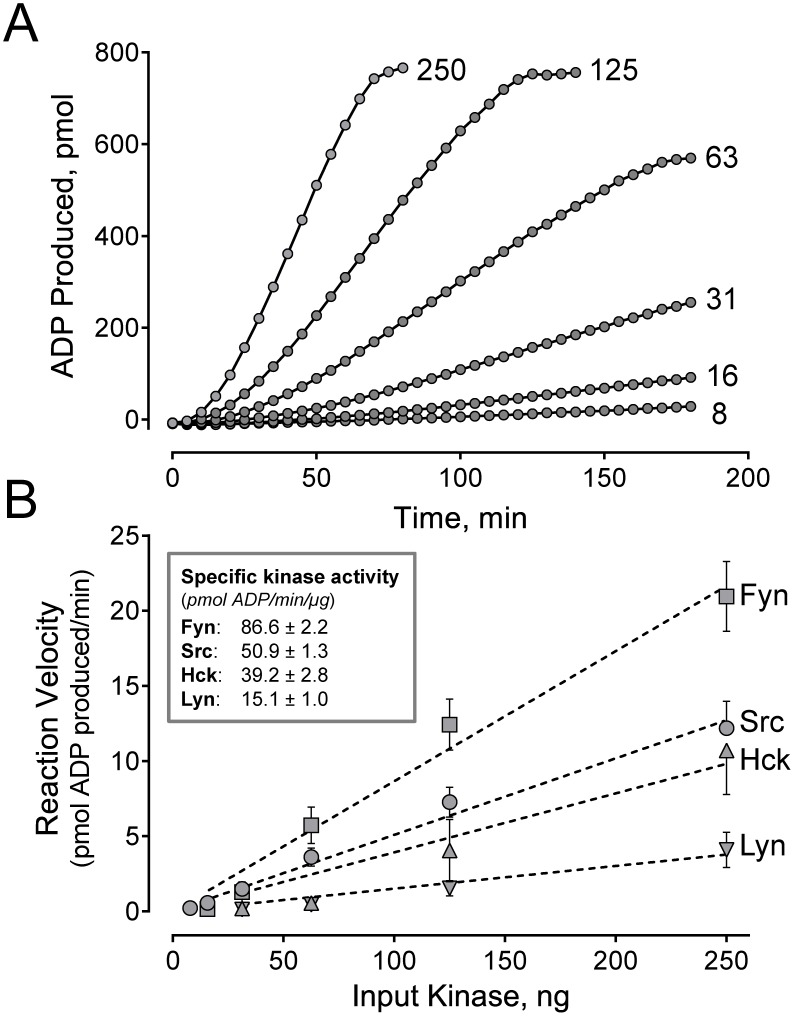
Linear relationship between SFK-YEEI activity and kinase protein input. A) Representative time course of ADP production for six concentrations of Src-YEEI. At higher kinase concentrations, the reaction rates plateau as the fluorescence reading reaches saturation. The linear portion of each curve was fit by linear regression analysis to provide the slope, which corresponds to the rate of the reaction in pmol ADP produced/min. B) Reaction rates for each SFK-YEEI protein are plotted against input kinase concentration. Curves were best-fit by linear regression analysis (dotted lines) and used to estimate the specific activity for each kinase (inset).

Prior to testing for SH3-based kinase activation using the VSL12 peptide, uniform assay conditions were established for each SFK. We first determined the K_m_ values for both ATP and the peptide substrate, YIYGSFK, for all four of the SFK-YEEI proteins using a kinetic assay. As shown in Figure S1, all of the SFKs obey Michaelis-Menten kinetics for both ATP and the peptide substrate. Table 2 summarizes the K_m_ values determined for each kinase. All four Src-family members showed very similar K_m_ values for ATP, ranging from 31 to 67 µM. These values are in the same range as those reported previously for these kinases, despite the use of diverse assay methods [24]–[26]. K_m_ values for the substrate peptide also varied by about two-fold, with Src-YEEI exhibiting the highest value (159 µM) and Fyn-YEEI the lowest (70 µM). To control for inherent variations in substrate utilization by each Src-family member, all subsequent experiments were done with the ATP and peptide substrate concentrations set to the respective K_m_ values for each kinase. Finally, kinase titrations were performed to determine the concentrations that yielded a basal rate of about 1 pmol ADP produced/min. Figure 4A shows the production of ADP over time for a range of six Src-YEEI concentrations. The reaction rate increases as a function of kinase concentration, as reflected in the increase in the slope of the linear portion of the curve for each kinase concentration. Reaction rates were determined in the same way for the remaining SFK-YEEI proteins, and the results are shown in Figure 4B. All four kinases showed a linear relationship between input kinase amount and reaction velocity over the range of kinase concentrations tested. Because the specific kinase activity differs for each family member, these experiments allowed us to identify input kinase amounts that yielded equivalent basal rates for subsequent SH3 domain-displacement experiments with VSL12.

**Table 2:** K_m_ Values for ATP and Peptide Substrate for Near-Full-Length SFKs.

Kinase	ATP (µM)	YIYGSFK (µM)
Src-YEEI	67.0±10.3	159.1±20.9
Fyn-YEEI	49.7±8.2	69.6±3.7
Hck-YEEI	55.8±18.5	83.8±12.5
Lyn-YEEI	31.1±9.70	83.2±4.9

The K_m_ values for ATP and the substrate peptide, YIYGSFK, were determined for each SFK-YEEI protein using the ADP Quest assay as described under Materials and Methods. ATP experiments were performed three times for each kinase and substrate experiments were performed four times for each kinase, except for Src-YEEI, where ATP experiments were performed twice and substrate experiments were performed three times. Mean values are shown for each kinetic constant ±S.E.

### Differential sensitivity of Src-family members to activation by VSL12 peptide binding

**Figure 5:**
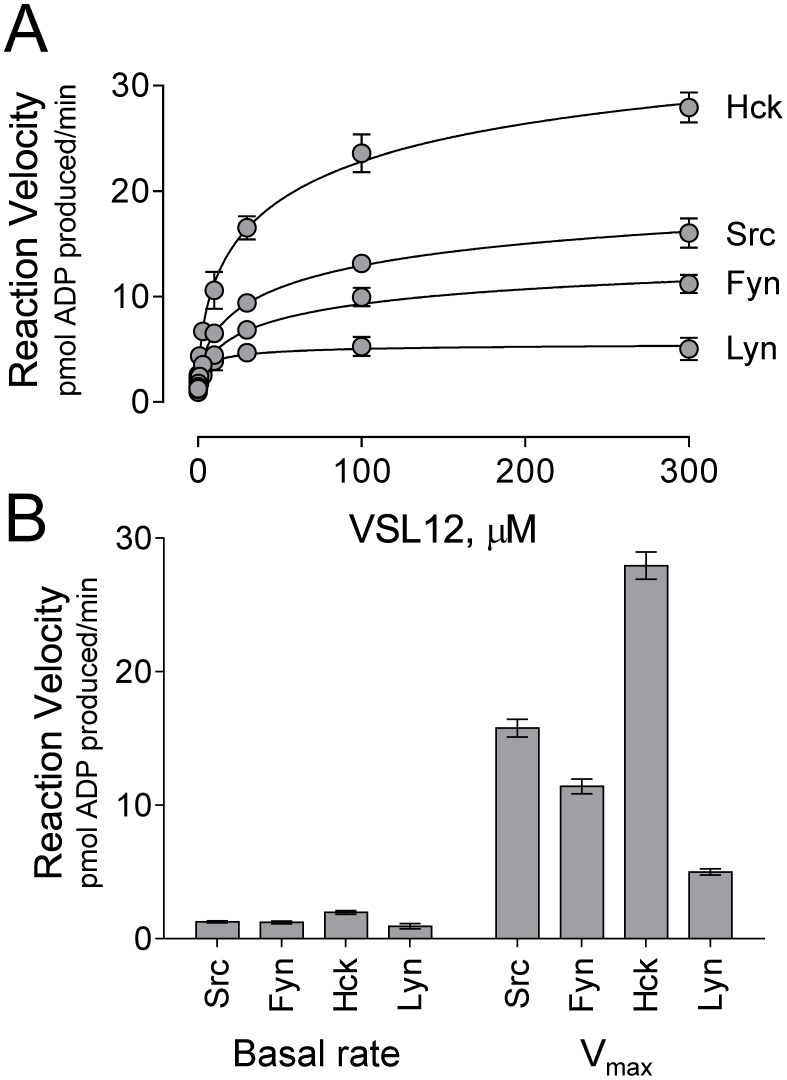
Differential sensitivity of individual SFK-YEEI proteins to activation by SH3 domain displacement. Each of the SFK-YEEI proteins shown was assayed in the presence of VSL12 over a range of concentrations (0.1 to 300 µM). ATP and substrate concentrations were set to the K_m_ for each kinase, and input kinase concentrations were set to achieve a basal reaction velocity of 1 pmol ADP produced/min. A) Each of the kinases is activated by VSL12 in a concentration-dependent manner. Plots of reaction velocity vs. VSL12 concentration were best-fit by the Michaelis-Menten equation, allowing for the determination of the V_max_. Each data point was assayed in triplicate and is shown as the mean ±S.E. B) Comparison of basal rate (left) and V_max_ (right) for each kinase in the presence of VSL12. Bars heights correspond to the mean values from triplicate experiments ±S.E.

We next tested the susceptibility of each Src-family member to SH3-based activation using the VSL12 peptide as an SH3 domain ‘agonist’. Using the established conditions for substrate, ATP, and kinase concentration as described above, the VSL12 peptide was titrated into the assay over a concentration range of 0.1 to 300 µM. Figure 5A shows the reaction velocity for each kinase as a function of VSL12 peptide concentration. These data show that all four Src-family members are susceptible to activation by the VSL12 peptide, providing strong evidence that SH3:linker interaction has an important role in kinase regulation across the entire Src family.

Data presented in Figure 5A demonstrate that the extent of kinase activation is saturable at higher concentrations of the VSL12 peptide. These data were therefore fit to Equation 1 (see Materials and Methods) to determine the V_max_ for each kinase reaction as a measure of the highest level of SH3-dependent activation attainable for each kinase. Figure 5B compares the basal reaction rate to the V_max_ for each kinase in the presence of VSL12. This analysis revealed that Src-family kinases have remarkably different responses to SH3-based kinase activation. Lyn-YEEI showed the smallest response, with a V_max_ of about 5 pmol ADP produced/min, while Hck-YEEI showed a much higher response, approaching 30 pmol ADP produced/min. Fyn-YEEI and Src-YEEI exhibited intermediate responses with values of 11 and 16 pmol ADP produced/min, respectively. These results support the idea that individual Src-family members have very different inherent enzymatic capacities, at least in response to SH3 domain-based activation.

In addition to defining the extent to which each kinase can be activated by VSL12 (V_max_), we also calculated an activation constant (K_act_) for each kinase in response to this SH3-binding peptide. K_act_ defines the concentration of VSL12 required to increase the reaction rate to one-half of the V_max_, and is calculated by subtracting the basal kinase activity (without VSL12) from the reaction rate observed at each VSL12 concentration and performing nonlinear regression analysis using Equation 2 (see Materials and Methods). As shown in Table 3, the K_act_ values for VSL12-based activation of Src-YEEI, Fyn-YEEI, and Hck-YEEI were all remarkably similar (22–25 µM). In contrast, Lyn-YEEI displayed a lower K_act_ compared to the other kinases (4 µM), which may suggest higher sensitivity to activation by SH3 domain displacement, although the maximum response observed was the lowest among the four kinases tested. Interestingly, Lyn and Hck are the most closely related among the SFKs tested in terms of phylogeny, yet their sensitivity to VSL12-induced activation is the most disparate.

**Table 3:** Basal Rate, Maximum Velocity, and Activation Constants for VSL12 with each SFK.

Kinase	Basal reaction velocity (pmol ADP produced/min)	V_max_ (pmol ADP produced/min)	K_act_ (µM)
Src-YEEI	1.27±0.08	15.77±0.67	22.42±2.23
Fyn-YEEI	1.22±0.10	11.41±0.55	24.95±1.91
Hck-YEEI	1.97±0.14	27.94±1.02	22.38±2.18
Lyn-YEEI	0.93±0.20	5.00±0.24	4.03±0.61

The basal reaction velocity, maximum velocity (V_max_), and activation constant (K_act_) were determined for each kinase in the ADP Quest assay as described under Materials and Methods. Basal velocity is the rate of kinase activation in the absence of the VSL12 peptide. Kinetic constants were determined in triplicate and are presented as the mean ±S.E.

### Hck and c-Src remain susceptible to activation by SH3 domain displacement after autophosphorylation

In addition to regulatory domain engagement, SFK activity is also regulated by the phosphorylation state of the kinase domain activation loop (Figure 1A). However, whether or not activation loop phosphorylation results in maximal kinase activation or whether the autophosphorylated kinase is still susceptible to further activation by SH3 domain displacement is not known. To explore this question, we focused on c-Src and Hck, because these are the only two family members for which crystal structures have been solved for the downregulated states [7]–[10]. Before examining the impact of VSL12 binding on SFK activity as a function of autophosphorylation, we first confirmed that the activation loops of recombinant Hck-YEEI and Src-YEEI were not phosphorylated prior to addition to the assay. To do this, the recombinant purified kinases were digested with pepsin and the phosphorylation states of the activation loop and tail tyrosines were determined by mass spectrometry. As shown in Figure S2, the C-terminal tail tyrosines are phosphorylated in both Src-YEEI and Hck-YEEI as expected for the inactive conformations, while no activation loop tyrosine phosphorylation was detected in either case. No unphosphorylated tail peptide was detected for either Src-YEEI or Hck-YEEI.

To test whether Src-YEEI and Hck-YEEI can be activated further by SH3 domain displacement after activation loop autophosphorylation, both kinases were preincubated in the presence or absence of ATP prior to testing for activation by VSL12. Time-course experiments revealed that kinase autophosphorylation reached a plateau after three hours of incubation with ATP (data not shown), so preincubation was conducted for this time period prior to assessment of the VSL12-induced response. The preincubated samples were subsequently analyzed by pepsin digestion and mass spectrometry to confirm phosphorylation of the activation loop (Figure S3). Activation loop phosphorylation of both Src-YEEI and Hck-YEEI was confirmed while the corresponding unphosphorylated peptides were not observed.

We observed a dramatic increase in the basal rate of Src-YEEI and Hck-YEEI kinase activity after pre-incubation with ATP. For Src-YEEI, the basal rate increased from 1.74 pmol to 6.38 ADP produced/min following ATP preincubation, while Hck-YEEI activity increased from 1.27 to 9.04 pmol ADP produced/min. This increase is presumably due to stabilization of the active site conformation as a result of autophosphorylation of the activation loop tyrosine. Note that the basal rate of each kinase following preincubation in the absence of ATP is consistent with the basal rates shown in Figure 5B and Table 3, indicating that the three hour preincubation period did not compromise kinase activity.

**Figure 6:**
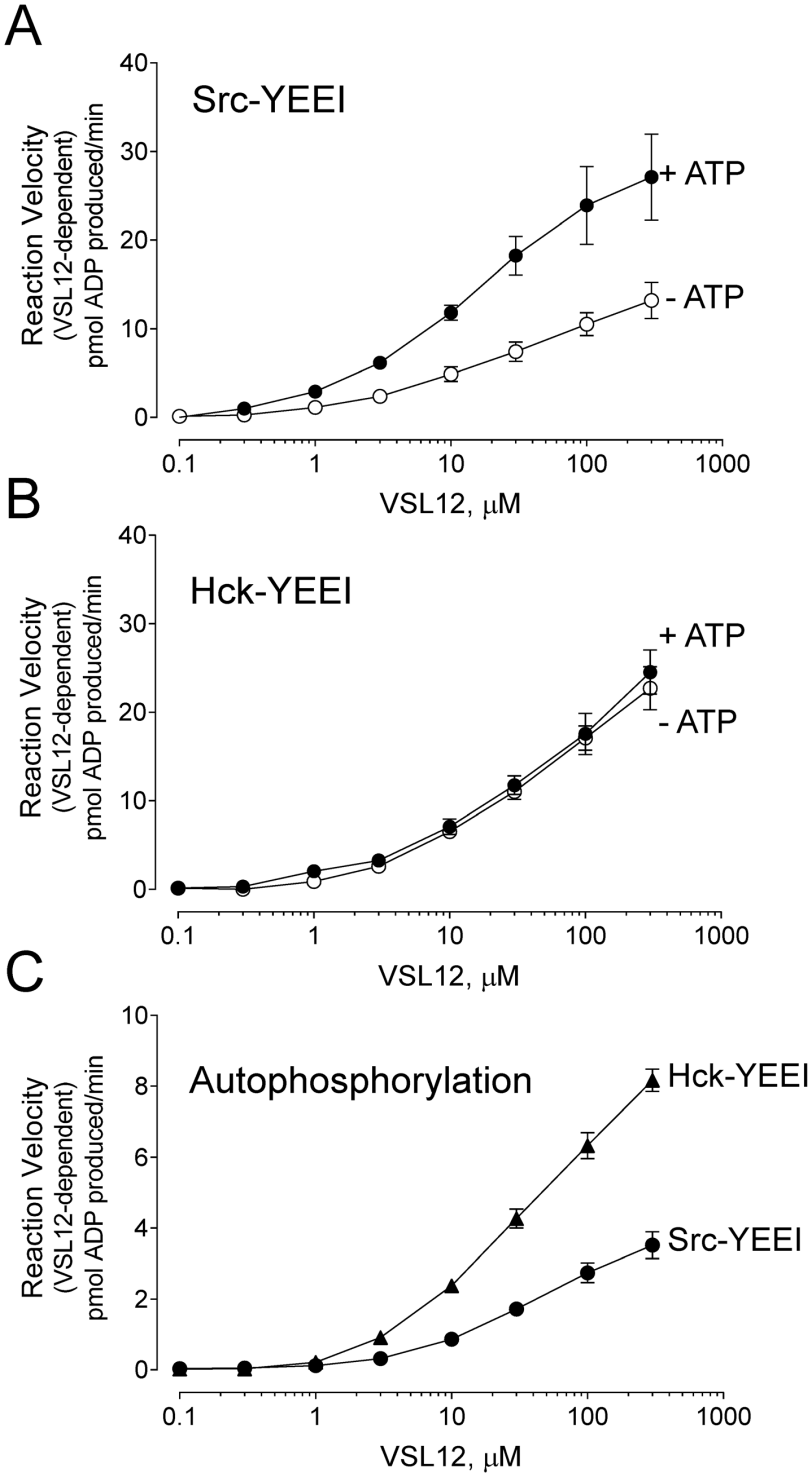
Src-YEEI and Hck-YEEI retain sensitivity to activation by SH3 domain displacement after autophosphorylation. Src-YEEI (A) and Hck-YEEI (B) were incubated in the absence or presence of ATP (at the K_m_) for 3 hours at 25°C to induce autophosphorylation of Tyr416 in the activation loop. The autophosphorylated kinases were then assayed for responsiveness to the SH3-binding peptide, VSL12. C) Autophosphorylation of Src-YEEI and Hck-YEEI was measured in response to the VSL12 peptide by running the kinase reactions in the absence of the substrate peptide. For all three panels, the basal reaction velocity (no VSL12) was subtracted from the rate at each VSL12 concentration, and the resulting linear reaction velocities were plotted as a function of VSL12 concentration as shown. Each data point was assayed in triplicate and the average value is plotted ±S.E.

The effects of SH3 domain displacement by VSL12 on Src-YEEI and Hck-YEEI activity as a function of autophosphorylation are shown in Figure 6A and 6B, respectively. In each case, the basal rate observed in the absence of VSL12 was subtracted from the rate at each peptide concentration to reveal additional activation resulting from VSL12 binding to the SH3 domain. Autophosphorylated Src-YEEI and Hck-YEEI were both activated by VSL12 in a concentration-dependent manner, demonstrating that phosphorylation of the activation loop does not uncouple the kinase domain from the regulatory influence of the SH3 domain. However, we observed remarkable differences in the responses of Src-YEEI vs. Hck-YEEI to VSL12 following preincubation with ATP. For Src-YEEI, preincubation with ATP enhanced the response to VSL12, indicating that autophosphorylation of Src-YEEI sensitizes the kinase domain to further activation by SH3 domain displacement. In contrast, the curves for Hck-YEEI activation by VSL12 were identical, whether the kinase was preincubated with ATP or not. This observation suggests that activation of Hck-YEEI by SH3 domain displacement is independent of activation loop phosphorylation. Alternatively, Hck-YEEI may autophosphorylate more rapidly than Src-YEEI, such that Hck-YEEI reaches maximum activation loop phosphorylation without ATP pre-incubation. To examine this possibility, we measured the effect of VSL12 on the rates of Hck-YEEI and Src-YEEI autophosphorylation by repeating the VSL12-activation reactions in the absence of the peptide substrate. Figure 6C shows that Hck-YEEI undergoes autophosphorylation more rapidly than Src-YEEI as a function of VSL12 input. This finding suggests that the autophosphorylation-dependent responses to VSL12 observed between Src-YEEI and Hck-YEEI are the result of inherent differences in autophosphorylation rates between these two Src family members. These differences also suggest that c-Src and Hck may have evolved to respond to different types of cellular inputs for activation that have important implications for signaling (see Discussion).

### Activation by VSL12 requires an intact SH3:linker interface in the downregulated kinases

**Figure 7:**
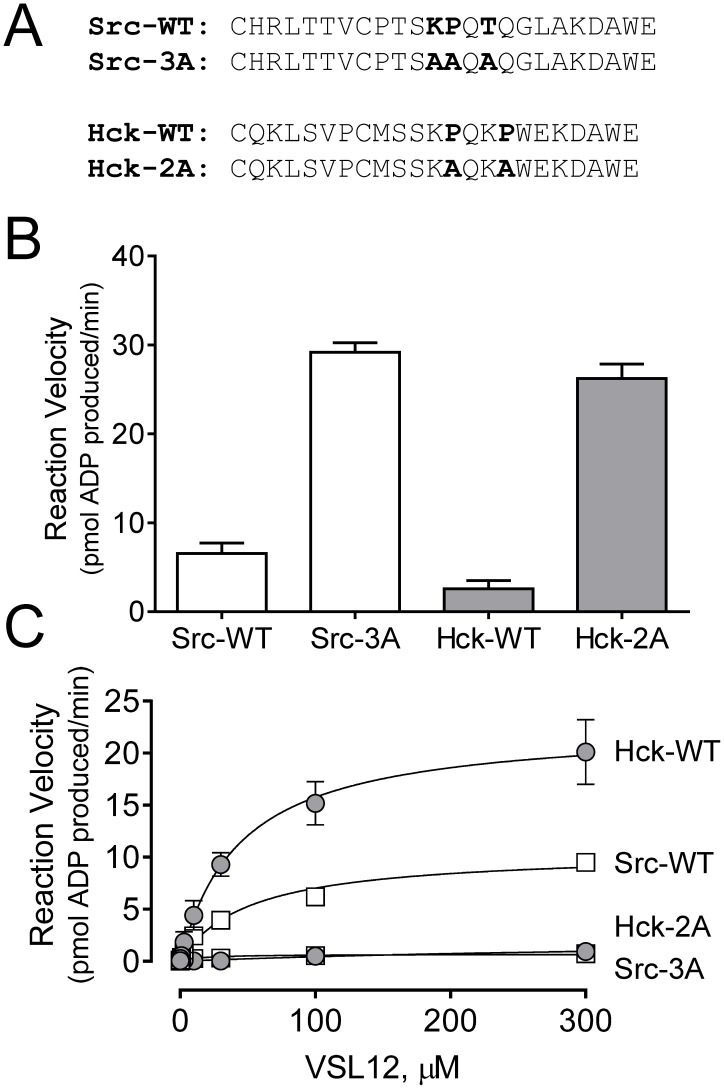
SFK linker mutants display higher basal kinase activity than their wild-type counterparts and are refractory to activation by VSL12. A) Sequences of the wild-type (WT) linkers of Src and Hck are shown. Residues involved in intramolecular engagement of the SH3 domain are highlighted in bold, and are replaced with alanines in the respective Src-3A and Hck-2A mutants as shown. B) Reaction velocities for equivalent amounts (125 ng/well) of Src-YEEI and Hck-YEEI with wild-type vs. mutant linkers were determined using the ADP Quest assay. Results are shown as the mean velocity for three replicate determinations ±S.E. C) Each of the SFK-YEEI proteins shown was assayed in the presence of VSL12 over a range of concentrations (0 to 300 µM). ATP and substrate concentrations were set to the K_m_ for each wild-type kinase, and input kinase concentrations were set to achieve a basal reaction velocity of 1 pmol ADP produced/min. Plots of reaction velocity vs. VSL12 concentration were best-fit by the Michaelis-Menten equation for the wild-type kinases, indicative of saturable activation kinetics by VSL12. Each data point was assayed in triplicate and is plotted as the mean velocity ±S.E.

Data presented in the previous sections suggest that the VSL12 peptide selectively interacts with downregulated SFKs through their SH3 domains, resulting in disruption of intramolecular SH3:linker interaction and subsequent kinase activation. Such a model predicts that SFK linker mutants should show higher basal kinase activity, and also be refractory to further activation by VSL12 binding. To test this idea, we created mutants of Hck-YEEI and Src-YEEI in which linker residues involved in SH3 binding and kinase downregulation are replaced with alanines (Figure 7A). Both mutants displayed higher basal kinase activity than their wild-type counterparts in the absence of the VSL12 peptide, consistent with loss of SH3 interaction with the modified linker (Figure 7B). Addition of VSL12 did not increase the activity of these mutants further (Figure 7C), consistent with the notion that VSL12 activates the wild-type kinases by disrupting SH3:linker interaction.

## Discussion

The mechanisms responsible for SFK downregulation have been established by extensive biochemical and structural studies and appear to be conserved among all family members (see Introduction). The precise mechanisms driving kinase activation, and whether or not they are also conserved across this kinase family, are less clear. Displacement of intramolecular regulatory interactions involving the SH2 and/or SH3 domains can cause kinase activation, suggesting that individual family members may be finely tuned to respond to inputs through their regulatory domains in specific ways. In this study, we provide direct evidence in support of this idea. Using a peptide ligand (VSL12) to induce kinase activation by SH3 domain displacement, we showed a wide range of sensitivities to this allosteric activation mechanism across the Src kinase family. Furthermore, we observed that c-Src becomes more susceptible to activation by SH3 domain displacement after autophosphorylation of the activation loop tyrosine. In contrast, activation of Hck through its SH3 domain appears to be independent of kinase domain autophosphorylation, revealing a very different response to this activating input despite the remarkable similarity to c-Src in terms of the structure of the inactive state [7]–[10].

We chose the VSL12 peptide as the SH3 ligand for these studies because previous work showed that it binds to the c-Src, Fyn, and Lyn SH3 domains with similar micromolar affinities [20], [27]. SPR results presented here show that VSL12 binds to the SH3 domains of these three SFKs as well as that of Hck with similar dissociation constants, providing a useful peptide ligand to compare the effect of SH3 domain displacement on kinase activity across the kinase family. The solution structure of the Src SH3 domain in complex with VSL12 reveals the key SH3 residues that interact with this peptide and suggests a mechanism for linker displacement [21]. The hydrophobic pocket formed by SH3 Tyr90 and Tyr136 is occupied by the lone proline residue in the Src SH2-kinase linker (Pro250; Figure 2B). This interaction is likely displaced by Leu11 and Pro12 in the VSL12 peptide, as these residues occupy the same pocket in the NMR structure. In addition, VSL12 Leu8 and Pro9 both make contacts with the SH3 domain, while the analogous positions are occupied by Thr252 and Gln253 in the linker; Gln253 does not contact the SH3 domain in the inactive c-Src structure (Figure 2B). Furthermore, VSL12 Arg6 forms a stabilizing ionic contact with conserved SH3 Asp99, and also makes a hydrogen bond with the side chain of Trp118. This interaction is missing in the SH3:linker interface in downregulated c-Src. The SH3 domain residues involved in VSL12 binding are conserved across the four SFKs studied here, consistent with the similarities in binding kinetics and affinities observed by SPR analysis (Table 1).

Work presented here supports the idea that Src-family kinases are uniformly susceptible to activation by SH3 domain displacement. In addition, VSL12-based activation of recombinant SFK proteins with YEEI tails suggests that disruption of SH2:tail interaction is not required for SH3-based activation, as the pYEEI sequence has a much higher affinity for the SH2 domain than the natural tail sequence [7], [28]. While the VSL12 peptide bound to and activated all four of the kinases tested, differences in the extent of activation were striking. Particularly surprising was the difference in sensitivity to SH3-based activation between Hck and Lyn, as they are the most similar in amino acid sequence among the entire Src kinase family. Our observation that Hck and Lyn demonstrate very different responses to SH3-based activation by VSL12 is consistent with previous reports with the Nef protein of HIV-1. Like VSL12, Nef binds to the SH3 domains of Hck and Lyn with similar affinity, yet Nef activates Hck more strongly both in vitro and in cell-based assays [29], [30]. One possibility is that maximal activation of Lyn may also require displacement of the C-terminal tail from the SH2 domain, which does not appear to be the case for Hck.

To understand how interaction with VSL12 induces kinase activation, the structures of Src-family kinases in the active vs. inactive conformations must be considered. In the downregulated structure of c-Src, the SH3 domain contacts both the SH2-kinase linker (as described above) and Arg318 in the N-lobe of the kinase domain (through SH3 Asp117). Another key residue known to couple the SH3-SH2 region to the kinase domain is Trp260 at the C-terminal end of the linker [31]. This conserved residue packs against helix C, helping to position it away from the active site and preventing formation of a salt bridge between Glu310 and Lys295 that is required for kinase function. Because VSL12 has a relatively high affinity for the SH3 domains of the SFKs, it is likely to bind to the SH3 domain, displace the linker and shift Trp260 away from the C helix. The stabilizing interaction between SH3 domain Asp117 and N-lobe Arg318 may also be disrupted as a consequence. Removal of these inhibitory constraints would allow the C helix to rotate inward, with Glu310 forming the critical salt bridge with Lys295. This rearrangement also exposes activation loop Tyr416 for autophosphorylation. Further support for this linker displacement mechanism in the activation of SFKs by VSL12 comes from studies of linker mutants. Alanine substitution of the key residues in the linker that pack against the SH3 domain in the downregulated structure results in constitutive activation of both Src-YEEI and Hck-YEEI. As a result, both kinases are refractory to further activation by VSL12.

Another remarkable difference between the SFKs relates to the coupling of activation loop autophosphorylation and sensitivity to SH3 domain displacement. Autophosphorylation of Tyr416 strongly stimulated both Hck and c-Src kinase activity in the absence of the VSL12 peptide. In addition, both kinases were further activated by VSL12 in a concentration-dependent manner, demonstrating that activation loop phosphorylation enhances kinase activity independently of regulatory domain displacement. This observation suggests that Tyr416 phosphorylation alone is sufficient to reorganize the active site for phosphotransfer. However, our data show that autophosphorylation alone does not maximally increase kinase activity, because addition of VSL12 enhanced both c-Src and Hck kinase activity to an even greater extent. The additional impact of this SH3 domain ligand on autophosphorylated SFKs implies that displacement of the SH3 domain relieves additional allosteric constraints on the kinase domain, possibly mediated through Trp260 or direct SH3 interaction with the N-lobe as described above. Furthermore, this observation implies that autophosphorylation of the activation loop does not cause release of the SH3 domain from the linker, at least for c-Src and Hck.

Our finding that autophosphorylated c-Src and Hck are still under the allosteric control of the SH3 domain is consistent with recent studies of c-Abl kinases. Like SFKs, the c-Abl kinase ‘core’ consists of a similar arrangement of SH3 and SH2 domains, an SH2-kinase linker, followed by the kinase domain. In this system, intramolecular interaction of the SH3 domain with the linker is also essential for downregulation of Abl kinase activity [32]. Interestingly, active mutants of c-Abl, as well as the constitutively active Bcr-Abl fusion protein associated with chronic myelogenous leukemia, are both inhibited by linker proline substitutions that enhance interaction with the SH3 domain [33]. Together with our data, these observations support the idea that activation loop phosphorylation and regulatory domain displacement represent independent modes of SFK and Abl regulation.

In summary, observations presented here suggest distinct regulatory controls for individual Src-family members, despite the fact that they are composed of the same component parts. These differences may have evolved to meet specific needs for kinase regulation under distinct physiological conditions. For example, c-Src is locally active in focal adhesions, where it regulates their turnover during cell adhesion and migration. One important mechanism of c-Src activation in focal contacts involves direct interaction with FAK. In this case, the SH3 and SH2 domains of c-Src interact with FAK, integrating the localization of c-Src to focal adhesions with kinase activation via regulatory domain displacement [13]. This interaction juxtaposes the c-Src and FAK kinase domains, raising the possibility of direct phosphorylation of the c-Src activation loop by FAK [34]. Maximal c-Src activation in focal adhesions may therefore require displacement of both regulatory domains plus trans-phosphorylation by another kinase, consistent with our findings. In contrast, Hck is expressed primarily in cells of innate immunity and is activated by diverse upstream signals including hematopoietic cytokines and F_c_ receptors [35]–[38]. In this case, receptor engagement with Hck through its SH3 and/or SH2 domains may be sufficient to induce a rapid and transient response, without the need for secondary phosphorylation on the activation loop. These intrinsic differences in the sensitivity of c-Src, Hck and other SFKs to activating signals may provide opportunities for discovery of selective small molecule probes of their functions.

## Materials and Methods

### Cloning, expression, and purification of recombinant SFK domains

The coding sequences for the isolated SH3 and SH2 domains of human c-Src, Fyn, Hck, and Lyn, and the isolated kinase domains (KD) of c-Src and Hck(corresponding to residues 81–142 (SH3), 143–244 (SH2), and 251–533 (KD) of c-Src, numbering according to PDB ID: 2SRC) were amplified by PCR and subcloned into the bacterial expression vector pET21a (EMD Millipore) using NdeI and XhoI restriction sites. The C-terminus of each construct was modified by the addition of the sequence LPHHHHHH as encoded by the XhoI site and the His_6_ tag in the vector. For expression of the SH3 and SH2 domains, *E. coli* strain Rosetta 2(DE3)pLysS (EMD Millipore) was transformed with each plasmid and cultures were grown at 37°C until the OD_600_ reached 0.8–1.0. Protein expression was induced with 0.4 mM isopropyl -D-thiogalactopyranoside (IPTG) for 4 h at 25°C. The isolated KDs were expressed as previously described by Seeliger, et al. [39] For purification, cell pellets were resuspended in ion-exchange start buffer (20 mM Tris-HCl, pH 8.5, 5 mM -mercaptoethanol, 10% glycerol), sonicated, and clarified by centrifugation. Soluble domain proteins were purified from the clarified lysates by anion-exchange (HiPrep Q FF; GE Healthcare), immobilized metal ion affinity (HiTrap Chelating HP; GE Healthcare), and size-exclusion chromatography (HiLoad 26/60 Superdex 75; GE Healthcare). Purified proteins were buffer-exchanged with HBS-EP (10 mM HEPES, pH 7.4, 150 mM NaCl, 3 mM EDTA, 0.05% v/v P20 surfactant) using Amicon Ultra-15 Centrifugal Filter Units with Ultracel-10 Membranes (EMD Millipore) for storage and surface plasmon resonance experiments. Recombinant purified protein concentrations were quantified by Bradford assay using the BioRad Protein Assay Dye Reagent Concentrate and purified BSA as a reference standard (Pierce).

### Expression and purification of recombinant SFK-YEEI proteins

Human Hck, Lyn, Fyn and c-Src clones were modified on their C-terminal tails to encode the sequence Tyr-Glu-Glu-Ile-Pro (YEEI variants) to enable autophosphorylation of the tail and high-yield purification in the down-regulated state without co-expression of Csk [7], [28]. In addition, the N-terminal unique domain of each kinase was replaced with a hexahistidine tag. The linker mutant proteins Src-3A-YEEI and Hck-2A-YEEI were produced using the QuikChange II XL Site-Directed Mutagenesis Kit (Agilent Technologies) using the Src-YEEI and Hck-YEEI cDNA clones, respectively, as templates. Each construct was used to produce a recombinant baculovirus in Sf9 insect cells using BaculoGold DNA and the manufacturer's protocol (BD Pharmingen) as previously described [23]. Src-YEEI, Src-3A-YEEI, and Hck-2A-YEEI were co-expressed with the *Yersinia pestis* YopH phosphatase to promote dephosphorylation of the activation loop tyrosine to help downregulate kinase activity [39], [40]. For these proteins, Sf9 cells were grown in a monolayer and infected with SFK-YEEI and YopH baculoviruses. Cells were harvested 72 h after infection for SFK-YEEI purification. All kinases were purified as previously described [23]. Purified proteins were stored in 20 mM Tris-HCl, pH 8.3, containing 100 mM NaCl. SFK-YEEI protein concentrations were determined by running dilutions of each recombinant protein on an SDS-PAGE gel together with dilutions of a BSA protein standard. Each gel was stained with Coomassie blue and scanned using a LICOR Odyssey system for densitometric determination of protein concentration.

### Surface Plasmon Resonance (SPR)

SPR experiments were performed using a Biacore 3000 Instrument (GE Healthcare) on a streptavidin (SA) biosensor chip. The high-affinity Src SH3-binding peptide VSLARRPLPPLP [20] was synthesized and biotinylated by the University of Pittsburgh Peptide Synthesis Core. The peptide was solubilized in HBS-EP buffer to a concentration of 5 nM, injected onto the SA surface at 10 l/min and immobilized to a level of 80 Response Units (RU). A biotinylated IB kinase substrate peptide, RHDSGLDSMKD (Enzo Life Sciences), was immobilized to 80 RU on the reference flow cells as a control for nonspecific binding of analytes to the peptide-SA surface. SFK SH3, SH2 and kinase domain proteins were injected in duplicate over a range of concentrations at 25°C at a flow rate of 30 l/min. Association was measured for 180 s, followed by 300 s of dissociation in HBS-EP running buffer. The chip surface was regenerated with HBS-EP buffer after each run. In addition to reference flow cell subtraction, HBS-EP buffer-only cycles were used to allow double referencing for all analyses. Binding curves were fit using a 1∶1 Langmuir binding model and the BIAevaluation software v. 4.1.1 (GE Healthcare) which was used to generate all kinetic data. To determine steady state K_D_ values, equilibrium responses were plotted against analyte concentrations, and the resulting curves were fit with the steady state model in the BIAevaluation software.

### Kinetic kinase assays

Kinetic kinase assays were performed using the ADP Quest Assay (DiscoveRx), which fluorimetrically monitors the production of ADP [41]. Briefly, the conversion of ATP to ADP is coupled to the production of pyruvate from phosphoenolpyruvate (PEP) by pyruvate kinase (PK). Pyruvate is converted to hydrogen peroxide by pyruvate oxidase, which in turn oxidizes Amplex Red to the fluorescent product, resorufin. Accumulation of ADP is measured as the increase in resorufin fluorescence at excitation and emission wavelengths of 530 nm and 590 nm, respectively. All assays were performed in quadruplicate in black 384-well microplates (Corning Catalog # 3571). ATP stocks (10 mM; Sigma) were prepared in 10 mM Tris-HCl, pH 7.0. The SFK substrate peptide (5 mM; sequence YIYGSFK; Anaspec) [42] was prepared in ADP Quest assay buffer (15 mM HEPES, pH 7.4, 20 mM NaCl, 1 mM EGTA, 0.02% Tween-20, 10 mM MgCl_2_, 0.1 mg/ml bovine -globulins). Kinase reactions were initiated by the addition of 5 l ATP to each well at 10 times the final concentration. The reactions were performed in a final assay volume of 50 l/well at 25°C. Assay plates were read at 5 min intervals for 3 h on a Molecular Devices SpectraMax M5 microplate reader.

### Substrate and ATP K_m_ determination in the ADP Quest assay

To measure the K_m_ for the YIYGSFK substrate peptide, the ATP concentration was held constant at 200 M, the maximum tolerated concentration in the assay. ATP is regenerated in the assay and is therefore not depleted as a function of substrate phosphorylation. Kinase concentrations were held constant at 150 ng/well for Src-YEEI, Fyn-YEEI, and Hck-YEEI, and 250 ng/well for Lyn-YEEI. The substrate peptide was titrated into the assay at various concentrations ranging from 1.95 to 500 M. To measure the K_m_ for ATP, the peptide substrate concentration was set to the K_m_ for each SFK, and kinase concentrations were held constant as stated above. ATP was titrated into the assay over a concentration range of 6.25 to 200 M. The resulting rate data were fit as described below.

### ADP Quest Data Analysis

Each ADP Quest assay included controls for non-enzymatic production of ADP and kinase autophosphorylation. Raw fluorescence data obtained from quadruplicate wells for each condition were averaged and corrected for the non-enzymatic rate of ADP production and SFK autophosphorylation. Corrected raw fluorescence units (RFU) were then plotted against time, in minutes, to determine the rate of each reaction. Linear regression analysis (GraphPad Prism) was performed on the linear portion of each corrected progress curve, and the slope yielded the reaction rate. Reaction rates were converted to pmol ADP produced/min using the correction factor 4.2 RU/pmol ADP, which was generated from a standard curve under identical reaction conditions. For ATP and substrate K_m_ experiments, plots of the reaction rates at each substrate or ATP concentration obeyed Michaelis-Menten kinetics and were best-fit by nonlinear regression analysis (GraphPad Prism). K_m_ values were determined using the equation,
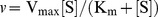
(1)where *v* =  the measured velocity, V_max_ =  the maximal reaction velocity, [S]  =  the substrate (peptide or ATP) concentration, and K_m_ =  the concentration of substrate or ATP at which the reaction velocity is half of the maximal velocity.

### SFK activation by the SH3 binding peptide in the ADP Quest Assay

To test each SFK for sensitivity to activation by VSL12, ATP and substrate concentrations were set to their respective K_m_ values for each SFK. Kinase concentrations were held constant for Src-YEEI (25 ng/well), Fyn-YEEI (30 ng/well), Hck-YEEI (80 ng/well), and Lyn-YEEI (120 ng/well), so as to yield a basal reaction rate of about 1 pmol ADP produced/min in each case. The VSL12 peptide was solubilized in ADP Quest assay buffer to 10 mM. The VSL12 peptide was titrated into the assay from 0.1 to 300 M. VSL12 peptide and kinase were pre-incubated for 15 min at 25°C before the addition of substrate, assay reagents, and ATP.

To test Src-YEEI and Hck-YEEI for sensitivity to activation by VSL12 after autophosphorylation, each kinase was pre-incubated in 550 l of ADP Quest assay buffer with or without ATP at the K_m_ for each kinase for 3 h at 25°C. Following pre-incubation, responsiveness to VSL12 activation was assayed as described above.

Kinase reaction rates at each VSL12 peptide concentration obeyed Michaelis-Menten kinetics and were best-fit by nonlinear regression. The V_max_ of each kinase reaction in the presence of VSL12 was determined using Equation 1. To calculate the activation constant (K_act_), the basal rate (rate in the absence of VSL12) was subtracted from the rate in the presence of VSL12 at each concentration. The transformed rates were plotted against VSL12 concentration. The resulting curves also obeyed Michaelis-Menten kinetics and were best-fit by nonlinear regression as per the method of Boerner, et al., 1996 [43]. K_act_ was calculated from
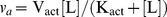
(2)where *v_a_* =  the measured velocity in the presence of VSL12 minus the velocity in the absence of VSL12, V_act_ =  the maximal reaction velocity in the presence of VSL12 minus the velocity in the absence of VSL12, [L]  =  the VSL12 concentration, and K_act_ =  the concentration of VSL12 at which the reaction velocity is half of the V_act_.

### Pepsin digestion and mass analysis

For the elucidation of kinase autophosphorylation sites, 50 pmol of Src-YEEI and Hck-YEEI were digested online with pepsin in potassium phosphate buffer (150 mM KH_2_PO_4_/150 mM K_2_HPO_4_, pH 2.5). The resulting peptides were separated using a Waters nanoAcquity UPLC system (Waters Corp, Milford, MA), trapped and desalted for 3 min at 100 µL/min and then separated in 8 min by an 8%–40% acetonitrile:water gradient at 40 µL/min. The separation column was a 1.0×100.0 mm Acquity UPLC C18 BEH (Waters) containing 1.7 µm particles. Mass spectra were obtained with a Waters Xevo-QTOF equipped with standard ESI source (Waters Corp., Milford, MA, USA). Mass spectra were acquired over an *m/z* range of 100 to 1900. Mass accuracy was ensured by calibration with 100 fmol/µL Glu-fibrinopeptide, and was less than 10 ppm throughout all experiments. Identification of the peptic fragments was accomplished through a combination of exact mass analysis and MS^E^ using custom IdentityE PLGS 2.5 Software from the Waters Corporation [44]. MS^E^ was performed by a series of low-high collision energies ramping from 5–30 V, therefore ensuring proper fragmentation of all the peptic peptides eluting from the LC system.

## Supporting Information

Figure S1 **Recombinant near-full-length Src-family kinases obey Michaelis-Menten kinetics.** Initial reaction velocities for each of the SFK-YEEI proteins shown were determined over a range of ATP and peptide substrate (sequence YIYGSFK) concentrations as described under Materials and Methods. Plots of reaction velocity vs. the concentration of ATP (*left panels*) and substrate (*right panels*) exhibited saturation kinetics and were fit to the Michaelis-Menten equation by non-linear regression analysis (GraphPad Prism Software). The resulting K_m_ and V_max_ values are presented in Table 2 in the main text.
(PDF)

Figure S2 **Mass spectrometric analysis of Src-YEEI and Hck-YEEI tail and activation loop peptides.** A) ESI-MS/MS spectra of Src-YEEI peptic peptide YFTSTESQpY^527^EEIP ([M+H]^+^ = 1683.68 Da), which is derived from the C-terminal tail, indicates that Tyr527 is phosphorylated (numbering as per crystal structure of c-Src; PDB ID: 2SRC). The mass difference between fragment ions b_8_ and b_9_ (blue) indicates the presence of a phosphate group attached to the tyrosine residue (243 Da). The y ions are colored in red. B) ESI-MS/MS spectra of Src-YEEI peptic peptide Y^416^TARQGAKF ([M+H]^+^ = 1041.54 Da), which maps to the activation loop in the kinase domain, indicates that Tyr416 is not phosphorylated (163 Da). The mass difference between fragment ions y_8_ and y_9_ (red) corresponds to the unphosphorylated tyrosine. C) ESI-MS/MS spectra of Hck-YEEI peptic peptide ESQpY^527^EEIP ([M+H]^+^ = 1074.40 Da), derived from the C-terminal tail, indicates that Tyr527 is phosphorylated. The mass difference between fragment ions b_3_ and b_4_ (blue) indicates the presence of a phosphate group attached to the tyrosine residue. D) ESI-MS/MS spectra of the Hck-YEEI peptic peptide Y^416^TAREGAKF ([M+H]^+^ = 1042.54 Da), derived from the activation loop, indicates that Tyr416 is not phosphorylated. The mass difference between fragment ions y_8_ and y_9_ (red) corresponds to the unphosphorylated tyrosine.
(PDF)

Figure S3 **Mass spectrometric analysis reveals quantitative phosphorylation of the Src-YEEI and Hck-YEEI activation loop tyrosines following preincubation with ATP.** Src-YEEI and Hck-YEEI were preincubated with ATP for 3 h followed by pepsin digestion and mass spectral analysis as described under Materials and Methods. A) ESI-MS/MS spectra of Src-YEEI peptic peptide IEDNEpY^416^TARQGAKF ([M+H]^+^ = 1721.75 Da), derived from the activation loop, indicates that Tyr416 is phosphorylated (numbering as per crystal structure of c-Src; PDB ID: 2SRC). The mass difference between fragment ions y_9_ and y_7_ (red) matches that of phosphotyrosine plus threonine for a total mass of 344 Da. B) ESI-MS/MS spectra of the Hck-YEEI peptic peptide ARVIEDNEpY^416^TARQGAKF ([M+H]^+^ = 2048.95 Da), derived from the activation loop, indicates that Tyr416 is phosphorylated. The mass difference between fragment ions y_8_ and y_9_ (red) corresponds to phosphotyrosine. Fragment b-series ions are also present (blue). We were unable to detect the corresponding unphosphorylated activation loop peptides in either spectrum, suggesting that preincubation with ATP under these conditions leads to stoichiometric phosphorylation of the activation loop.
(PDF)

## References

[1] Parsons SJ, Parsons JT (2004) Src family kinases, key regulators of signal transduction. Oncogene 23: 7906–7909. 15489908 10.1038/sj.onc.1208160

[2] Thomas SM, Brugge JS (1997) Cellular functions regulated by Src family kinases. Annu Rev Cell Dev Biol 13: 513–609. 9442882 10.1146/annurev.cellbio.13.1.513

[3] Yeatman TJ (2004) A renaissance for SRC. Nat Rev Cancer 4: 470–480. 15170449 10.1038/nrc1366

[4] Resh MD (1999) Fatty acylation of proteins: new insights into membrane targeting of myristoylated and palmitoylated proteins. Biochim Biophys Acta 1451: 1–16. 10446384 10.1016/s0167-4889(99)00075-0

[5] Boggon TJ, Eck MJ (2004) Structure and regulation of Src family kinases. Oncogene 23: 7918–7927. 15489910 10.1038/sj.onc.1208081

[6] Chong YP, Ia KK, Mulhern TD, Cheng HC (2005) Endogenous and synthetic inhibitors of the Src-family protein tyrosine kinases. Biochim Biophys Acta 1754: 210–220. 16198159 10.1016/j.bbapap.2005.07.027

[7] Schindler T, Sicheri F, Pico A, Gazit A, Levitzki A, et al (1999) Crystal structure of Hck in complex with a Src family-selective tyrosine kinase inhibitor. Mol Cell 3: 639–648. 10360180 10.1016/s1097-2765(00)80357-3

[8] Xu W, Doshi A, Lei M, Eck MJ, Harrison SC (1999) Crystal structures of c-Src reveal features of its autoinhibitory mechanism. Mol Cell 3: 629–638. 10360179 10.1016/s1097-2765(00)80356-1

[9] Sicheri F, Moarefi I, Kuriyan J (1997) Crystal structure of the Src family tyrosine kinase Hck. Nature 385: 602–609. 9024658 10.1038/385602a0

[10] Xu W, Harrison SC, Eck MJ (1997) Three-dimensional structure of the tyrosine kinase c-Src. Nature 385: 595–602. 9024657 10.1038/385595a0

[11] Briggs SD, Smithgall TE (1999) SH2-kinase linker mutations release Hck tyrosine kinase and transforming activities in rat-2 fibroblasts. J Biol Chem 274: 26579–26583. 10473622 10.1074/jbc.274.37.26579

[12] Brown MT, Cooper JA (1996) Regulation, substrates, and functions of Src. Biochim Biophys Acta 1287: 121–149. 8672527 10.1016/0304-419x(96)00003-0

[13] Thomas JW, Ellis B, Boerner RJ, Knight WB, White GC, et al (1998) SH2- and SH3-mediated interactions between focal adhesion kinase and Src. J Biol Chem 273: 577–583. 9417118 10.1074/jbc.273.1.577

[14] Moarefi I, LaFevre-Bernt M, Sicheri F, Huse M, Lee C-H, et al (1997) Activation of the Src-family tyrosine kinase Hck by SH3 domain displacement. Nature 385: 650–653. 9024665 10.1038/385650a0

[15] Briggs SD, Sharkey M, Stevenson M, Smithgall TE (1997) SH3-mediated Hck tyrosine kinase activation and fibroblast transformation by the Nef protein of HIV-1. J Biol Chem 272: 17899–17902. 9218412 10.1074/jbc.272.29.17899

[16] Meyn MA III, Smithgall TE (2009) Chemical genetics identifies c-Src as an activator of primitive ectoderm formation in murine embryonic stem cells. Sci Signal 2: ra64. 19825829 10.1126/scisignal.2000311 PMC2775445

[17] Anneren C, Cowan CA, Melton DA (2004) The Src family of tyrosine kinases is important for embryonic stem cell self-renewal. J Biol Chem 279: 31590–31598. 15148312 10.1074/jbc.M403547200

[18] Zhang X, Meyn MA III, Smithgall TE (2013) c-Yes Tyrosine Kinase Is a Potent Suppressor of ES Cell Differentiation and Antagonizes the Actions of Its Closest Phylogenetic Relative, c-Src. ACS Chem Biol 9: 139–146. 23895624 10.1021/cb400249b PMC3875617

[19] Sancier F, Dumont A, Sirvent A, Paquay de PL, Edmonds T, et al (2011) Specific oncogenic activity of the Src-family tyrosine kinase c-Yes in colon carcinoma cells. PLoS One 6: e17237. 21390316 10.1371/journal.pone.0017237 PMC3044743

[20] Rickles RJ, Botfield MC, Zhou XM, Henry PA, Brugge JS, et al (1995) Phage display selection of ligand residues important for Src homology 3 domain binding specificity. Proc Natl Acad Sci U S A 92: 10909–10913. 7479908 10.1073/pnas.92.24.10909 PMC40540

[21] Feng S, Kasahara C, Rickles RJ, Schreiber SL (1995) Specific interactions outside the proline-rich core of two classes of Src homology 3 ligands. Proc Natl Acad Sci U S A 92: 12408–12415. 8618911 10.1073/pnas.92.26.12408 PMC40367

[22] Lerner EC, Trible RP, Schiavone AP, Hochrein JM, Engen JR, et al (2005) Activation of the Src Family Kinase Hck without SH3-Linker Release. J Biol Chem 280: 40832–40837. 16210316 10.1074/jbc.M508782200

[23] Trible RP, Emert-Sedlak L, Smithgall TE (2006) HIV-1 Nef selectively activates SRC family kinases HCK, LYN, and c-SRC through direct SH3 domain interaction. J Biol Chem 281: 27029–27038. 16849330 10.1074/jbc.M601128200 PMC2892265

[24] Boerner RJ, Barker SC, Knight WB (1995) Kinetic mechanisms of the forward and reverse pp60c-src tyrosine kinase reactions. Biochemistry 34: 16419–16423. 8845369 10.1021/bi00050a024

[25] Barker SC, Kassel DB, Weigl D, Huang X, Luther MA, et al (1995) Characterization of pp60c-src tyrosine kinase activities using a continuous assay: autoactivation of the enzyme is an intermolecular autophosphorylation process. Biochemistry 34: 14843–14851. 7578094 10.1021/bi00045a027

[26] Knight ZA, Shokat KM (2005) Features of selective kinase inhibitors. Chem Biol 12: 621–637. 15975507 10.1016/j.chembiol.2005.04.011

[27] Rickles RJ, Botfield MC, Weng Z, Taylor JA, Green OM, et al (1994) Identification of Src, Fyn, Lyn, PI3K and Abl SH3 domain ligands using phage display libraries. EMBO J 13: 5598–5604. 7988556 10.1002/j.1460-2075.1994.tb06897.x PMC395523

[28] Porter M, Schindler T, Kuriyan J, Miller WT (2000) Reciprocal regulation of Hck activity by phosphorylation of Tyr(527) and Tyr(416). Effect of introducing a high affinity intramolecular SH2 ligand. J Biol Chem 275: 2721–2726. 10644735 10.1074/jbc.275.4.2721

[29] Saksela K, Cheng G, Baltimore D (1995) Proline-rich (PxxP) motifs in HIV-1 Nef bind to SH3 domains of a subset of Src kinases and are required for the enhanced growth of Nef^+^ viruses but not for down-regulation of CD4. EMBO J 14: 484–491. 7859737 10.1002/j.1460-2075.1995.tb07024.x PMC398106

[30] Briggs SD, Lerner EC, Smithgall TE (2000) Affinity of Src family kinase SH3 domains for HIV Nef in vitro does not predict kinase activation by Nef in vivo. Biochemistry: 489–495. 10.1021/bi992504j 10642173

[31] LaFevre-Bernt M, Sicheri F, Pico A, Porter M, Kuriyan J, et al (1998) Intramolecular regulatory interactions in the Src family kinase Hck probed by mutagenesis of a conserved tryptophan residue. Journal of Biological Chemistry 273: 32129–32134. 9822689 10.1074/jbc.273.48.32129

[32] Hantschel O, Superti-Furga G (2004) Regulation of the c-Abl and Bcr-Abl tyrosine kinases. Nat Rev Mol Cell Biol 5: 33–44. 14708008 10.1038/nrm1280

[33] Panjarian S, Iacob RE, Chen S, Wales TE, Engen JR, et al (2013) Enhanced SH3/linker interaction overcomes Abl kinase activation by gatekeeper and myristic acid binding pocket mutations and increases sensitivity to small molecule inhibitors. J Biol Chem 288: 6116–6129. 23303187 10.1074/jbc.M112.431312 PMC3585049

[34] Mitra SK, Schlaepfer DD (2006) Integrin-regulated FAK-Src signaling in normal and cancer cells. Curr Opin Cell Biol 18: 516–523. 16919435 10.1016/j.ceb.2006.08.011

[35] Wang AV, Scholl PR, Geha RS (1994) Physical and functional association of the high-affinity immunoglobulin G receptor (Fc gamma RI) with the kinases Hck and Lyn. J Exp Med 180: 1165–1170. 8064233 10.1084/jem.180.3.1165 PMC2191633

[36] Kedzierska K, Vardaxis NJ, Jaworowski A, Crowe SM (2001) FcgammaR-mediated phagocytosis by human macrophages involves Hck, Syk, and Pyk2 and is augmented by GM-CSF. J Leukoc Biol 70: 322–328. 11493626

[37] Durden DL, Kim HM, Calore B, Liu Y (1995) The Fc gamma RI receptor signals through the activation of Hck and MAP kinase. J Immunol 154: 4039–4047. 7535819

[38] Bhattacharjee A, Pal S, Feldman GM, Cathcart MK (2011) Hck is a key regulator of gene expression in alternatively activated human monocytes. J Biol Chem 286: 36709–36723. 21878628 10.1074/jbc.M111.291492 PMC3196116

[39] Seeliger MA, Young M, Henderson MN, Pellicena P, King DS, et al (2005) High yield bacterial expression of active c-Abl and c-Src tyrosine kinases. Protein Sci 14: 3135–3139. 16260764 10.1110/ps.051750905 PMC2253236

[40] Iacob RE, Pene-Dumitrescu T, Zhang J, Gray NS, Smithgall TE, et al (2009) Conformational disturbance in Abl kinase upon mutation and deregulation. Proc Natl Acad Sci U S A 106: 1386–1391. 19164531 10.1073/pnas.0811912106 PMC2635808

[41] Charter NW, Kauffman L, Singh R, Eglen RM (2006) A generic, homogenous method for measuring kinase and inhibitor activity via adenosine 5′-diphosphate accumulation. J Biomol Screen 11: 390–399. 16751335 10.1177/1087057106286829

[42] Lam KS, Wu J, Lou Q (1995) Identification and characterization of a novel synthetic peptide substrate specific for Src-family protein tyrosine kinases. Int J Pept Protein Res 45: 587–592. 7558590 10.1111/j.1399-3011.1995.tb01323.x

[43] Boerner RJ, Kassel DB, Barker SC, Ellis B, DeLacy P, et al (1996) Correlation of the phosphorylation states of pp60c-src with tyrosine kinase activity: the intramolecular pY530-SH2 complex retains significant activity if Y419 is phosphorylated. Biochemistry 35: 9519–9525. 8755732 10.1021/bi960248u

[44] Plumb RS, Johnson KA, Rainville P, Smith BW, Wilson ID, et al (2006) UPLC/MS(E); a new approach for generating molecular fragment information for biomarker structure elucidation. Rapid Commun Mass Spectrom 20: 1989–1994. 16755610 10.1002/rcm.2550

