# Supplementary material for: Differential Sensitivity of Src-Family Kinases to Activation by SH3 Domain Displacement
Source: PLoS One. 2014 Aug 21;9(8):e105629. doi: 10.1371/journal.pone.0105629 (PMC4140816; doi:10.1371/journal.pone.0105629)
Supplement: Figure S1 — Recombinant near-full-length Src-family kinases obey Michaelis-Menten kinetics. Initial reaction velocities for each of the SFK-YEEI proteins shown were determined over a range of ATP and peptide substrate (sequence YIYGSFK) concentrations as described under Materials and Methods. Plots of reaction velocity vs. the concentration of ATP (left panels) and substrate (right panels) exhibited saturation kinetics and were fit to the Michaelis-Menten equation by non-linear regression analysis (GraphPad Prism Software). The resulting Km and Vmax values are presented in Table 2 in the main text. (PDF) [file pone.0105629.s001.pdf]

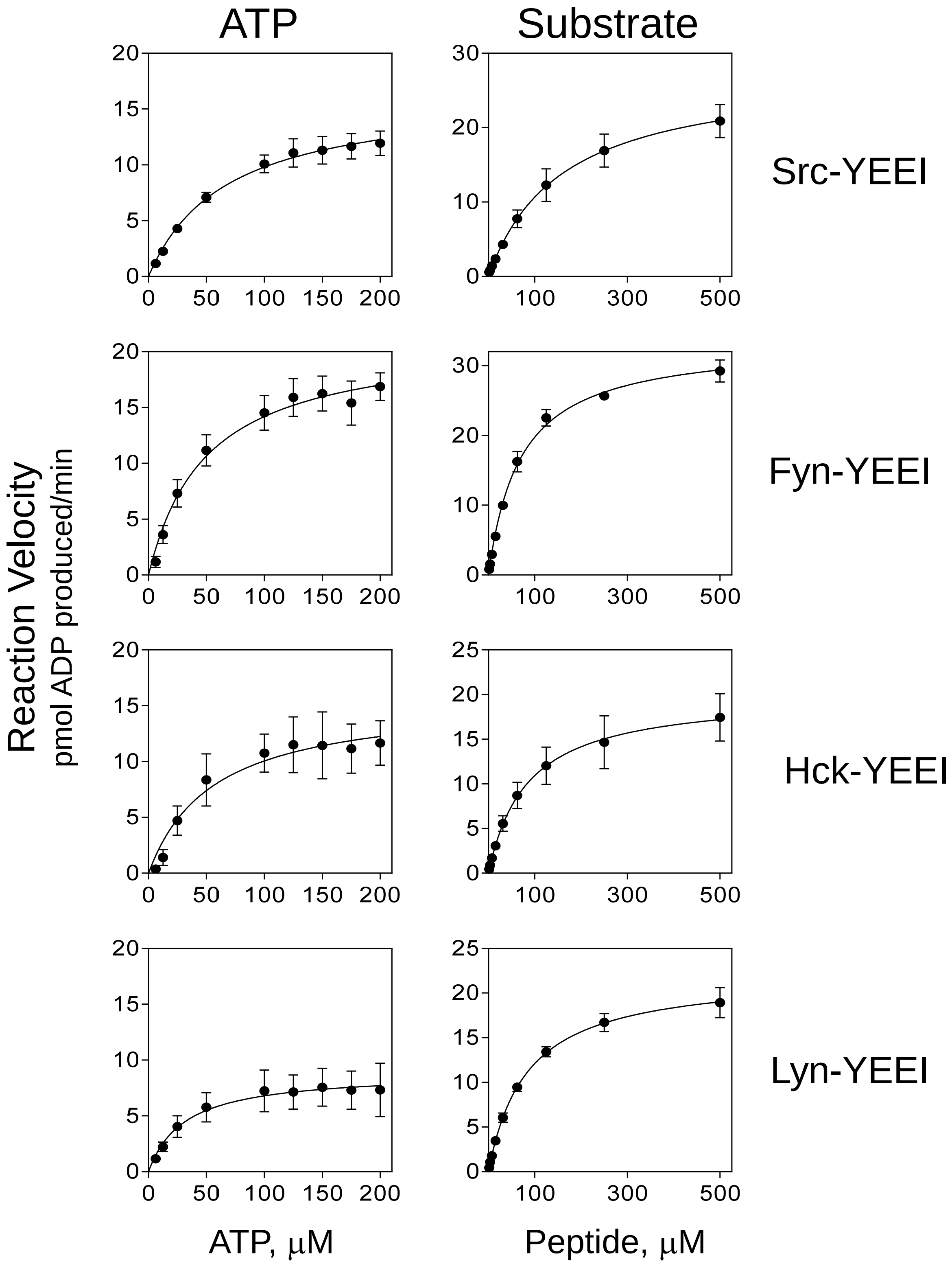

Figure S1. Recombinant near-full-length Src-family kinases obey Michaelis-Menten kinetics. Initial reaction velocities for each of the SFK-YEEI proteins shown were determined over a range of ATP and peptide substrate (sequence YIYGSFK) concentrations as described under Materials and Methods. Plots of reaction velocity vs. the concentration of ATP (*left panels*) and substrate (*right panels*) exhibited saturation kinetics and were fit to the Michaelis-Menten equation by non-linear regression analysis (GraphPad Prism Software). The resulting K_m and V_{max} values are presented in Table 2 in the main text.
