# Supplementary material for: Differential Sensitivity of Src-Family Kinases to Activation by SH3 Domain Displacement
Source: PLoS One. 2014 Aug 21;9(8):e105629. doi: 10.1371/journal.pone.0105629 (PMC4140816; doi:10.1371/journal.pone.0105629)
Supplement: Figure S2 — Mass spectrometric analysis of Src-YEEI and Hck-YEEI tail and activation loop peptides. A) ESI-MS/MS spectra of Src-YEEI peptic peptide YFTSTESQpY527EEIP ([M+H]+ = 1683.68 Da), which is derived from the C-terminal tail, indicates that Tyr527 is phosphorylated (numbering as per crystal structure of c-Src; PDB ID: 2SRC). The mass difference between fragment ions b8 and b9 (blue) indicates the presence of a phosphate group attached to the tyrosine residue (243 Da). The y ions are colored in red. B) ESI-MS/MS spectra of Src-YEEI peptic peptide Y416TARQGAKF ([M+H]+ = 1041.54 Da), which maps to the activation loop in the kinase domain, indicates that Tyr416 is not phosphorylated (163 Da). The mass difference between fragment ions y8 and y9 (red) corresponds to the unphosphorylated tyrosine. C) ESI-MS/MS spectra of Hck-YEEI peptic peptide ESQpY527EEIP ([M+H]+ = 1074.40 Da), derived from the C-terminal tail, indicates that Tyr527 is phosphorylated. The mass difference between fragment ions b3 and b4 (blue) indicates the presence of a phosphate group attached to the tyrosine residue. D) ESI-MS/MS spectra of the Hck-YEEI peptic peptide Y416TAREGAKF ([M+H]+ = 1042.54 Da), derived from the activation loop, indicates that Tyr416 is not phosphorylated. The mass difference between fragment ions y8 and y9 (red) corresponds to the unphosphorylated tyrosine. (PDF) [file pone.0105629.s002.pdf]

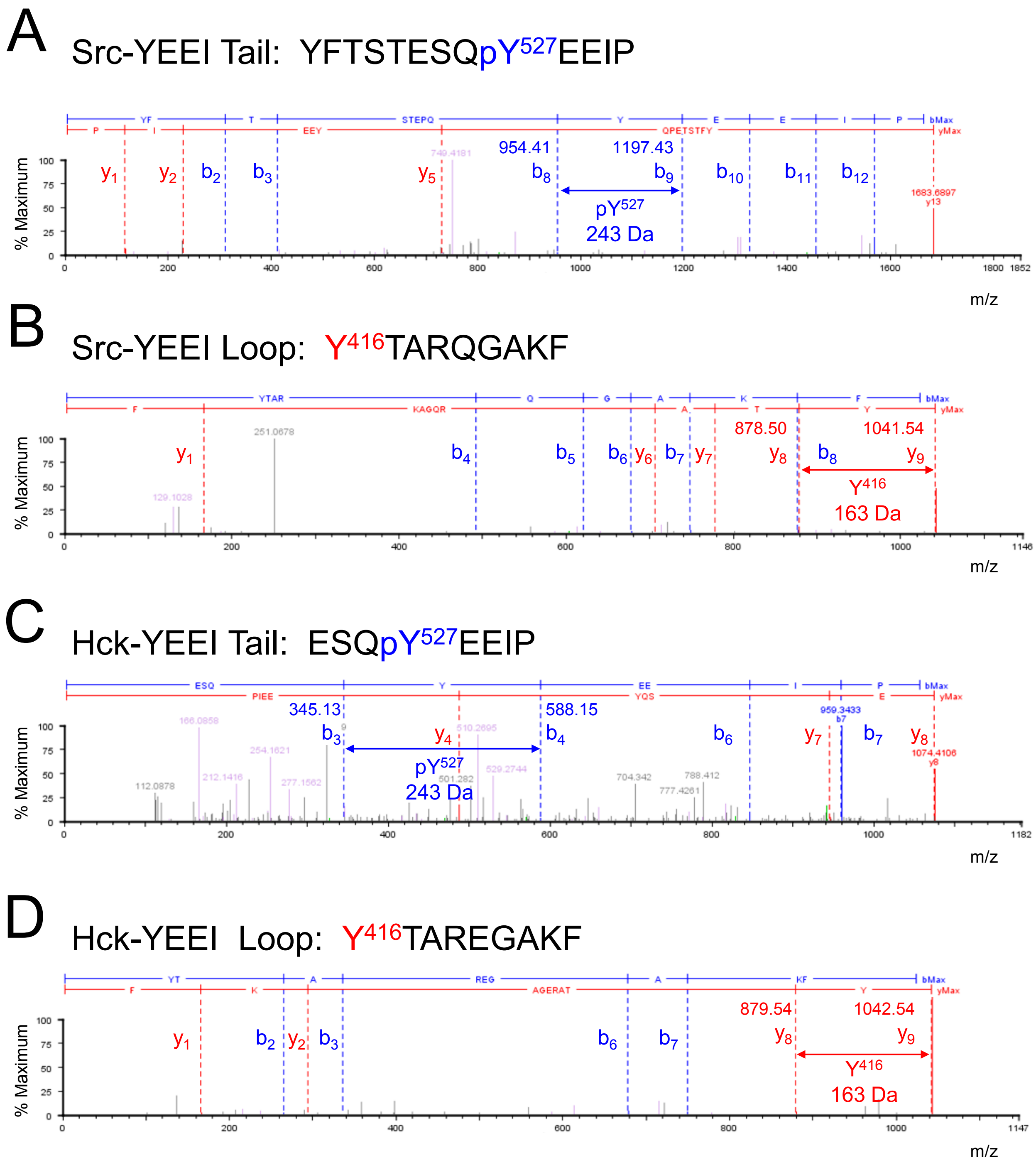

Figure S2. Mass spectrometric analysis of Src-YEEI and Hck-YEEI tail and activation loop peptides. A) ESI-MS/MS spectra of Src-YEEI peptic peptide YFTSTESQpY^{527}EEIP ($[M+H]^+$ = 1683.68 Da), which is derived from the C-terminal tail, indicates that Tyr527 is phosphorylated (numbering as per crystal structure of c-Src; PDB ID: 2SRC). The mass difference between fragment ions b_8 and b_9 (blue) indicates the presence of a phosphate group attached to the tyrosine residue (243 Da). The y ions are colored in red. B) ESI-MS/MS spectra of Src-YEEI peptic peptide Y^{416}TARQGAKF ($[M+H]^+$ = 1041.54 Da), which maps to the activation loop in the kinase domain, indicates that Tyr416 is not phosphorylated (163 Da). The mass difference between fragment ions y_8 and y_9 (red) corresponds to the unphosphorylated tyrosine. C) ESI-MS/MS spectra of Hck-YEEI peptic peptide ESQpY^{527}EEIP ($[M+H]^+$ = 1074.40 Da), derived from the C-terminal tail, indicates that Tyr527 is phosphorylated. The mass difference between fragment ions b_3 and b_4 (blue) indicates the presence of a phosphate group attached to the tyrosine residue. D) ESI-MS/MS spectra of the Hck-YEEI peptic peptide Y^{416}TAREGAKF ($[M+H]^+$ = 1042.54 Da), derived from the activation loop, indicates that Tyr416 is not phosphorylated. The mass difference between fragment ions y_8 and y_9 (red) corresponds to the unphosphorylated tyrosine.
