# Supplementary material for: Differential Sensitivity of Src-Family Kinases to Activation by SH3 Domain Displacement
Source: PLoS One. 2014 Aug 21;9(8):e105629. doi: 10.1371/journal.pone.0105629 (PMC4140816; doi:10.1371/journal.pone.0105629)
Supplement: Figure S3 — Mass spectrometric analysis reveals quantitative phosphorylation of the Src-YEEI and Hck-YEEI activation loop tyrosines following preincubation with ATP. Src-YEEI and Hck-YEEI were preincubated with ATP for 3 h followed by pepsin digestion and mass spectral analysis as described under Materials and Methods. A) ESI-MS/MS spectra of Src-YEEI peptic peptide IEDNEpY416TARQGAKF ([M+H]+ = 1721.75 Da), derived from the activation loop, indicates that Tyr416 is phosphorylated (numbering as per crystal structure of c-Src; PDB ID: 2SRC). The mass difference between fragment ions y9 and y7 (red) matches that of phosphotyrosine plus threonine for a total mass of 344 Da. B) ESI-MS/MS spectra of the Hck-YEEI peptic peptide ARVIEDNEpY416TARQGAKF ([M+H]+ = 2048.95 Da), derived from the activation loop, indicates that Tyr416 is phosphorylated. The mass difference between fragment ions y8 and y9 (red) corresponds to phosphotyrosine. Fragment b-series ions are also present (blue). We were unable to detect the corresponding unphosphorylated activation loop peptides in either spectrum, suggesting that preincubation with ATP under these conditions leads to stoichiometric phosphorylation of the activation loop. (PDF) [file pone.0105629.s003.pdf]

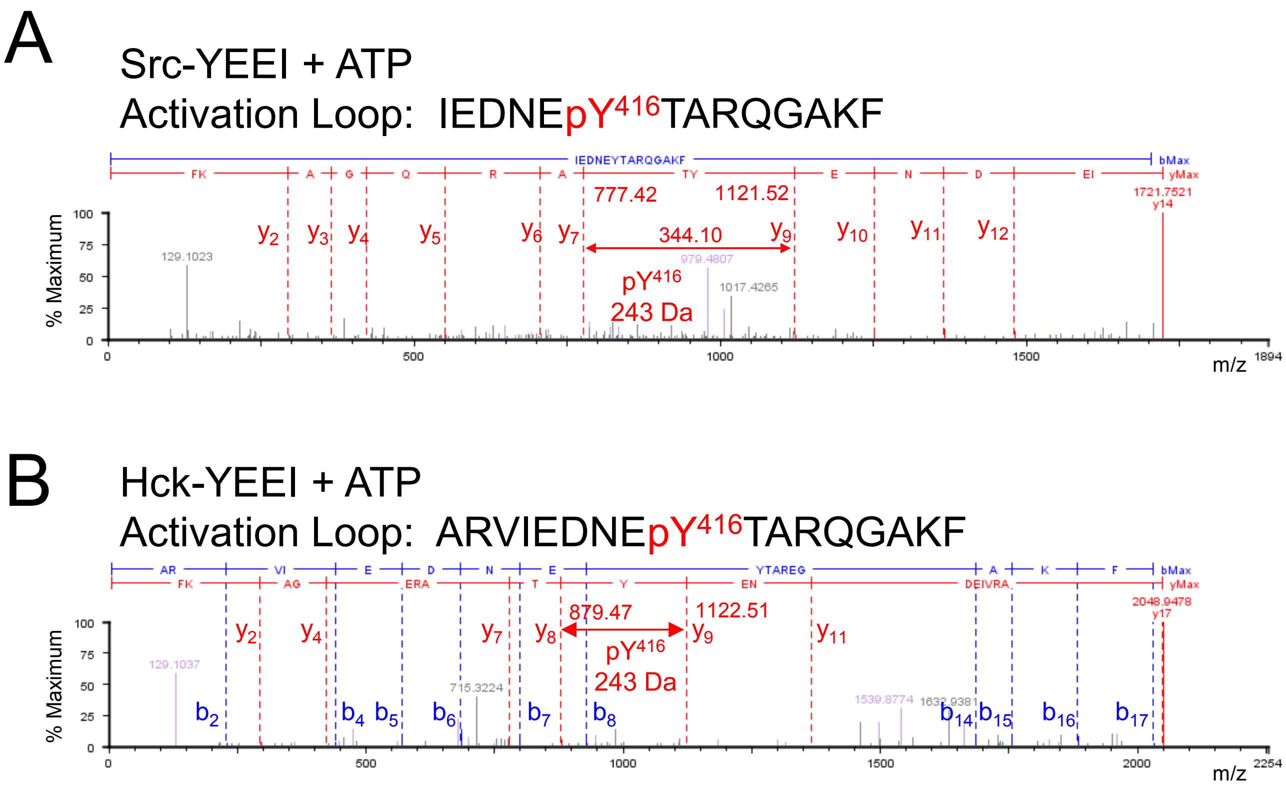

Figure S3. Mass spectrometric analysis reveals quantitative phosphorylation of the Src-YEEI and Hck-YEEI activation loop tyrosines following preincubation with ATP. Src-YEEI and Hck-YEEI were preincubated with ATP for 3 h followed by pepsin digestion and mass spectral analysis as described under Materials and Methods. A) ESI-MS/MS spectra of Src-YEEI peptic peptide IEDNEpY^{416}TARQGAKF ($[M+H]^+$ = 1721.75 Da), derived from the activation loop, indicates that Tyr416 is phosphorylated (numbering as per crystal structure of c-Src; PDB ID: 2SRC). The mass difference between fragment ions y_9 and y_7 (red) matches that of phosphotyrosine plus threonine for a total mass of 344 Da. B) ESI-MS/MS spectra of the Hck-YEEI peptic peptide ARVIEDNEpY^{416}TARQGAKF ($[M+H]^+$ = 2048.95 Da), derived from the activation loop, indicates that Tyr416 is phosphorylated. The mass difference between fragment ions y_8 and y_9 (red) corresponds to phosphotyrosine. Fragment b-series ions are also present (blue). We were unable to detect the corresponding unphosphorylated activation loop peptides in either spectrum, suggesting that preincubation with ATP under these conditions leads to stoichiometric phosphorylation of the activation loop.
